# The recent advances, drawbacks, and the future directions of CMRI in the diagnosis of IHD

**DOI:** 10.1038/s41598-021-94311-4

**Published:** 2021-07-22

**Authors:** Moram A. Fagiry, Ikhlas Abdelaziz, Rob Davidson, Mustafa Z. Mahmoud

**Affiliations:** 1 Diagnostic Radiologic Technology Department, College of Medical Radiological Sciences, Sudan University of Science and Technology, PO Box 1908, Zip Code: 11111 Khartoum, Sudan; 2 Department of Medical Imaging and Radiation Sciences, College of Applied Medical Sciences, University of Jeddah, Jeddah, Saudi Arabia; 3 Faculty of Health, University of Canberra, Canberra, ACT Australia; 4 Radiology and Medical Imaging Department, College of Applied Medical Sciences, Prince Sattam bin Abdulaziz University, Al-Kharj, Saudi Arabia

**Keywords:** Cardiology, Diseases, Medical research

## Abstract

Ischemic heart disease (IHD), also known as coronary artery disease (CAD), is a leading cause of morbidity and mortality in adults. The aims of this research were to study the recent advances on the prognostic and diagnostic value, drawbacks, and the future directions of cardiac magnetic resonance imaging (CMRI) in the diagnosis of IHD. One hundred patients with IHD who had been clinically diagnosed were enrolled in this study prospectively. CMRI; Siemens Magnetom Sola 1.5 T MRI scanner was used to examine the patients. To confirm the diagnosis, conventional coronary angiography was used. CMRI revealed that the left ventricular (LV) volumes and systolic function of male and female patients differed by age decile were 28.9 ± 3.5%; 32 ± 1.7%, 53.3 ± 11.2; 58 ± 6.6 ml, 100.6 ± 7.1; 98.3 ± 14.7 bpm, 5.4 ± 1.4; 5.8 ± 1.5 L/min, 189 ± 14.3; 180 ± 10.9 ml, and 136 ± 3.1; 123 ± 4.4 ml for the left ventricle ejection fraction (LVEF), stroke volume (SV), heart rate, cardiac output, end diastolic volume (EDV), and end systolic volume (ESV), respectively. CMRI has sensitivity, specificity, and accuracy of 97%, 33.33%, and 95.15%, respectively. Finally, CMRI provides a comprehensive assessment of LV function, myocardial perfusion, and viability, as well as coronary anatomy.

## Introduction

Ischemic heart disease (IHD), also known as coronary artery disease (CAD), can be accurately diagnosed with advanced radio-diagnostic techniques such as cardiac catheterization and computed tomography cardiac angiography (CTCA). However, such applications are associated with a number of limitations that have an impact on patient safety and the accuracy of diagnoses. The problem with both cardiac catheterization and CTCA is that the patient is exposed to a high radiation dose during the procedure. As a result, using cardiac magnetic resonance (CMR) technologies to overcome such issues is extremely beneficial to the patient^1–8^. As a result, the purpose of this study was to investigate the utility of cardiac magnetic resonance imaging (CMRI) as a diagnostic tool for IHD. This study's specific objectives are to update the reader on the current state of CMRI, with a special emphasis on the basic CMR sequences in IHD. Recent advances in the prognostic and diagnostic value of CMR in the evaluation of IHD are also discussed. Furthermore, the limitations of CMRI in the evaluation of IHD in the study samples will be presented in this study.

## Material and methods

### Study design and patient selection

One hundred patients with IHD were enrolled prospectively in this study after providing informed written and verbal consent from a local ethics committee at the Radiology and Medical Imaging Department, King Fahad Medical City (KFMC), Riyadh-Saudi Arabia, that was obtained prior to the study's start. Informed consent was obtained from parents or legal guardians of participants who are under the age of 18. The local ethics committee (LEC) of the College of Medical Radiological Sciences (CMRS), Sudan University of Science and Technology, Khartoum, Sudan approved study (Ethical approval number: CMRS LEC 94/2019). The ethics procedures used were in accordance with the 1975 Helsinki Declaration, which was updated in 2013. Patients who had been clinically examined by a referring cardiologist and were scheduled for CMRI and conventional coronary angiography were included in the study after their consent was obtained. CMRI was used on patients to localize the affected coronary artery and predict the affected myocardial segments. Conventional coronary angiography was the gold standard tool used to confirm the diagnosis in all patients. Other inclusion criteria included sinus heart rhythm, the ability to hold one's breath for 10–20 Sec, and normal serum creatinine. Exclusion criteria included hemodynamic instability, atrial fibrillation, MRI contraindications, claustrophobia, pacemaker or metal implants, contrast material contraindications, including known allergy, and renal insufficiency (serum creatinine greater than 1.4 mg/dl).

### Equipment

A Siemens Magnetom Sola 1.5 T MRI scanner with a superconducting magnet (Siemens Healthineers AG, Erlangen, Germany) was used. In the current study, we followed Abdelrahman et al.^9^ guidelines, our patients were examined in the supine position, head first using a respiratory sensor and ECG gating. Additionally, a sensitivity encoding (SENSE) cardiac coil was used.

**Figure 1:**
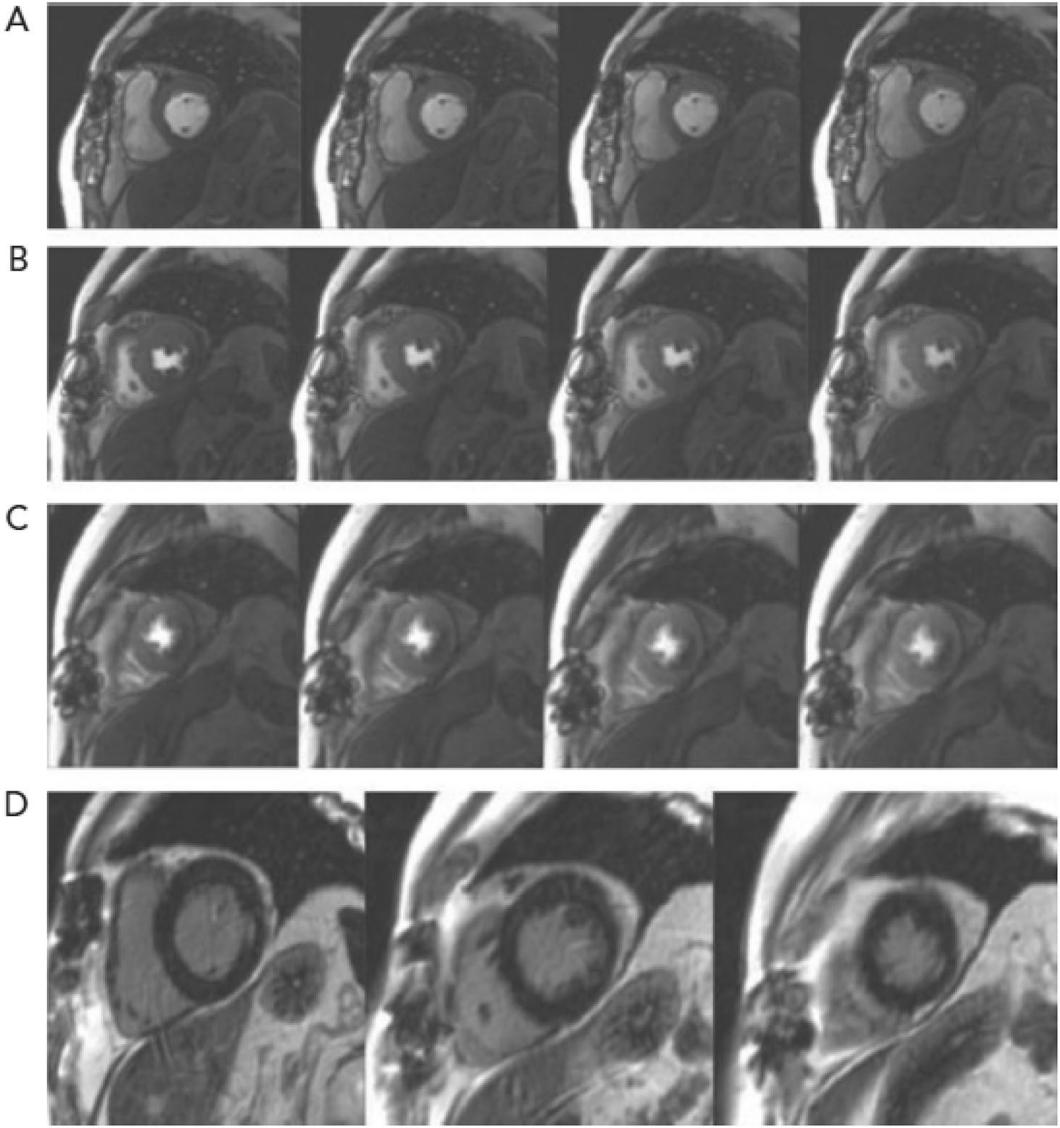
CMRI shows perfusion in four consecutive cardiovascular cycles in the short axis slices in the base (**A**), mid (**B**), and apical left ventricular portions (**C**). Panel (**D**) shows late gadolinium enhancement pictures in similar sections. There is significant ischemia in the inferior and inferolateral wall^10^.

For the contrast agent, all patients were cannulated with a wide bore intravenous line. The patients were subjected to a standard magnetic resonance examination, which included the following procedures: (i) Scout images were collected in orthogonal orientations to aid in the planning of the final long-axis and short-axis views. (ii) Before beginning the actual index test, perform a first pass rest perfusion imaging with scan angle geometry identical to that of the short axis cine views to carefully exclude any wraparound or trigger artifacts. This included an intravenous bolus injection of 0.025 mmol/kg gadopentetate dimeglumine (Magnevist) at a rate of 5 mL/s was followed by a flush of 20 mL of saline solution at the same rate. Scanning began approximately 10 Sec after contrast injection began and lasted approximately 1 min. A hybrid gradient echo-planar imaging pulse sequence was used to perform breath-hold first-pass perfusion MRI. This pulse sequence produces three sections (at the basal, mid-cavitary, and apical levels) (Fig. 1) in the short-axis view, covering the entire left ventricle every other heart beat, with the following parameters: Repetition time/echo time (TR/TE): 2.9/1.46, field of view (FOV): 350 × 350 mm^2^, phases: 25, number of signal averages (NSA): 1, matrix: 128 × 128, bandwidth: 125 kHz, flip angle: 200, scan time: 1 Sec, slice thickness: 8 mm, slice number: 3. (iii) An additional bolus of 0.2 mmol/kg gadopentetate dimeglumine was administered immediately following the end of the rest perfusion scan. (iv) Functional cine images were obtained in short axis view using an electrocardiographic gated, breath hold balanced fast field echo (b-FFE) sequence. During repeated breath-holds, a stack of eight to eleven short-axis views were obtained, starting from the mitral valve insertion and covering the entire left ventricle with the following parameters: TR/TE: 4.4/2.5, FOV: 300 × 300 mm^2^, phases: 25, NSA: 1, matrix: 128 × 128, slice thickness: 8 mm, slice number: 8–11. This sequence was carried out between the previously mentioned injection of an additional bolus of contrast and the delayed gadolinium enhancement sequences. (v) Standard delayed gadolinium enhancement imaging was performed using segmental inversion recovery balanced turbo field echo (IR-b-TFE) 10–15 min after the last intravenous bolus was injected. With the following parameters, contrast-enhanced images were acquired in the short axis plane and at least one of the long axis planes: TR/TE: 3.8/1.86, FOV: 300 × 300 mm^2^, inversion time (TI): 260–350, NSA: 1, matrix: 128 × 128, bandwidth: 125 kHz, flip angle: 15°, scan time: 9–15 Sec, slice thickness: 8 mm, slice number: 8–11^10^. The mean time of the CMRI imaging examination was about 30–35 min.

Results of CMRI in the current study were subdivided into two groups the same as in the study of Abdelrahman et al.^9^, into: (i) Viable myocardial segment was defined as having no myocardial scarring or having ≤ 50% myocardial scarring. Subendocardial scarring was defined as 25% myocardial scarring and partial thickness scarring as 50% myocardial scarring. (ii) A nonviable myocardial segment was defined, as scarring that was > 50% of the thickness of the myocardium. On delayed-enhancement imaging, a no-reflow zone (microvascular obstruction) was visible as a dark region surrounded by hyperenhancing myocardium and/or a first pass resting perfusion defect corresponding to delayed myocardial enhancement. The results of the CMRI were compared to the results of the corresponding conventional coronary angiograms in this study.

### CMRI technique in IHD

Adequate patient preparation prior to a CMRI examination is a required component of good CMRI practice. The following were some general pointers for patient preparation: (i) Consider a single-shot module or free breathing with real-time image acquisition if patient has trouble with breath holding, arrhythmia, or motion artifacts. (ii) Consider using an abdominal band to reduce artifacts in cases of difficulty due to extensive respiratory motion. (iii) In cases of difficulties due to pericardial effusion and a weak ECG signal, considered a peripheral pulse gating. (iv) If there are difficulties due to ghost artifacts caused by pleural effusion and respiratory difficulties, considered postponing the CMRI imaging until after pleural effusion drainage. These IHD patient preparation guidelines for CMRI were consistent with the Asian Society of Cardiovascular Imaging (ASCI) standardized practice protocol for cardiac magnetic resonance imaging^11^. CMRI sequences used in IHD patients in the current study (Table 1) were consistent with the Korean Society of Cardiovascular Imaging (KOSCI) practical and standard CMRI protocol and guideline for CMRI^12^.

**Table 1:** CMRI sequences in IHD patients using Siemens (1.5 Tesla) scanner.

Module	Sequence	Siemens (1.5 Tesla) scanner
Morphology imaging	Black or dark blood imaging	HASTE
	T_1_, T_2_ IR or Triple IR	TSE BB
Cine imaging	Bright blood cine and cine tagging	Cine True FISP
	SSFP or FFE gradient echo	
Perfusion imaging	PWI, TSI, EPI	EPI
LGE imaging	IR GRE or SSFP, PSIR	IR TurboFLASH
Flow imaging	Velocity-encoded cine imaging	PC

Black blood *BB*, Echo planar imaging *EPI*, Fast field echo-steady state free precession *FFE-SSFP*, Fast imaging with steady state precession *FISP*, Fast low angle shot *FLASH*, Gradient echo *GRE*, Half-Fourier acquisition single-shot turbo spin-echo *HASTE*, Inversion recovery *IR*, Late gadolinium enhancement *LGE*, Phase contrast *PC*, Phase-sensitive inversion-recovery *PSIR*, Perfusion weighted image *PWI*, Turbo spin echo *TSE*, Time-signal intensity *TSI.*

CMRI was performed using the precise planes required for imaging. MRI technologists in this study were familiar with various image axes for accurate imaging interpretation, despite the fact that recent MRI units provide a support system for the CMRI plane. The imaging plane of CMRI in IHD patients used in this study was consistent with the KOSCI practical and standard CMRI protocol and guideline for CMRI^12^.

CMRI analysis was performed with a personal computer and semiautomated software (CMRtools, Cardiovascular Imaging Solutions, London, UK). The analysis consisted of three major steps: First, in all cardiac phases, the LV endocardial and epicardial borders were delineated in all planes; second, the systolic descent and twist of the mitral valve was calculated by tracking valve motion on the long axis cines and used to correct for systolic LV volume loss due to atrioventricular ring descent; and third, blood pool thresholding was used to distinguish the papillary muscles. The LV mass was calculated using end-systolic frames. The LV volume/time curve generated from all frames of all cines was used to calculate end-systolic (ESV) and end-diastolic (EDV) volumes, and there was no requirement to choose the largest and smallest ventricular frames. The difference between EDV and ESV was used to calculate stroke volume (SV), and EF was calculated as SV/EDV. When measuring mass (equivalent to weighing the LV), papillary muscles were included, but not when measuring volume (equivalent to blood pool techniques). The longitudinal atrioventricular plane descent was measured in the septum and lateral wall and expressed as a ventricular length ration. Finally, the end-diastolic and end-systolic sphericity indexes were calculated. The diastolic function was calculated from the time/volume curve's derivative and expressed as a peak filling rate (PFR). The ratio, as well as the early and active peak filling rates (PFR_E_ and PFR_A_), were calculated^13^. CMRI at 1.5 T necessitates protocol optimization, careful shimming, and RF pulse adjustment to avoid artifacts. Furthermore, when using Gadolinium-based MRI contrast agents, it is critical to reduce the relaxation times of nuclei within the body. These considerations and patient safety applied in this study were similar to the considerations and patient safety of CMRI in IHD patients stated in the KOSCI guideline for CMRI^12^.

Cine imaging, perfusion imaging, LGE imaging, flow imaging, morphological imaging, tissue characterization T_1_, T_2_, and T_2_ star (T2*) mapping, and coronary angiography are among the CMRI exam modules used in this study in IHD patients. Table 2 summarizes the purpose of each exam module. These scanning modules were compatible with the exam modules outlined in the KOSCI CMRI guideline^12^.

**Table 2:** Demonstrates purpose of CMRI exam modules in IHD patients.

No	CMRI exam modules in IHD patients	Purpose
1	Cine imaging	Assess cardiac wall motion
2	Perfusion imaging	Evaluate myocardial perfusion (ischemia)
3	Late gadolinium enhancement (LGE) imaging	Evaluate myocardial viability
4	Flow imaging	Measure flow velocity and volume
5	Morphology imaging	Delineate anatomic structures
6	Tissue characterization T_1_ mapping	Evaluate the absolute T_1_ value of the myocardium
7	Tissue characterization T_2_ mapping	Evaluate the absolute T_2_ value of the myocardium
8	Tissue characterization T_2_* mapping	Evaluate the absolute T_2_* value of the myocardium and assess cardiac iron deposition in diseases such as thalassemia major
9	Coronary angiography	Evaluate coronary artery disease

### Statistical analysis

The Statistical Package for the Social Sciences version 20 for Windows (IBM Corporation, Armonk, NY, USA) was used to analyze the data, and the results were initially summarized in the form of comparison tables and graphs. All study variables were presented as mean ± SD because the obtained data had a symmetric normal distribution. The Student's *t*-test (unpaired *t*-test) was used to compare variables. The significance of the findings was determined using the *P*-value. A *p* ≤ 0.05 value was considered significant. The binomial test was used to compare observed frequencies of symptoms and lifestyle risk factors in IHD patients to a given probability parameter (by default, the probability parameter was 0.5). To determine the significance of the findings, the test was performed with a one-tailed alternative. A statistical diagnostic test was performed on the study samples to determine the sensitivity, specificity, accuracy, positive and negative likelihood ratio, and positive and negative predictive value of CMRI in the diagnosis of IHD. The probability that a test result will be positive when the disease is present is referred to as sensitivity (true positive rate). The probability that a test result will be negative when the disease is not present is referred to as specificity (true negative rate). The overall probability that a patient is correctly classified is referred to as accuracy. The positive likelihood ratio is the ratio of the likelihood of a positive test result given the presence of the disease to the likelihood of a positive test result given the absence of the disease. The negative likelihood ratio is the ratio of the likelihood of a negative test result in the presence of the disease to the likelihood of a negative test result in the absence of the disease. Finally, positive predictive value (PPV) is the probability that the disease exists when the test is positive, whereas negative predictive value (NPV) is the probability that the disease does not exist when the test is negative.

## Results

One hundred (61% males and 39% females; mean age of 50 ± 20.1 and 35 ± 18.4 for males and females, respectively) patients with IHD were enrolled prospectively in this study after providing written and verbal consent and receiving approval from the local ethics committee at the Radiology and Medical Imaging Department, KFMC, Riyadh, Saudi Arabia (Table 3). CMRI was used on all of them to localize the affected coronary artery and predict the affected myocardial segments. Conventional coronary angiography was the gold standard tool used to confirm the diagnosis in all patients.

**Table 3:** Age range (years), age distribution (n; %), and mean age (mean ± SD) in the IHD patients.

Age ranges (years)	Male distribution (n; %)	Female distribution (n; %)	Male mean age (mean ± SD)	Female mean age (mean ± SD)
13–23	(11; 18%)	(4; 10.3%)	(21 ± 3.4)	(19.5 ± 2.4)
24–34	(7; 11.5%)	(3; 7.7%)	(27 ± 3.0)	(29 ± 2.9)
35–45	(8; 13.1%)	(10; 25.6%)	(38 ± 1.3)	(38.5 ± 4.2)
46–56	(16; 26.2%)	(4; 10.3%)	(53 ± 3.1)	(51 ± 3.4)
57–67	(11; 18%)	(14; 35.9%)	(63 ± 3.1)	(60.5 ± 2.1)
68–78	(4; 6.6%)	(1; 2.6%)	(75 ± 2.1)	(71 ± 2.2)
79–89	(3; 4.9%)	(2; 5.0%)	(85 ± 0.6)	(81 ± 0.9)
90–100	(1; 1.6%)	(1; 2.6%)	(91 ± 0.0)	(93 ± 0.0)
Total	(61; 100%)	(39; 100%)	(50 ± 20.1)	(35 ± 18.4)

**Figure 2:**
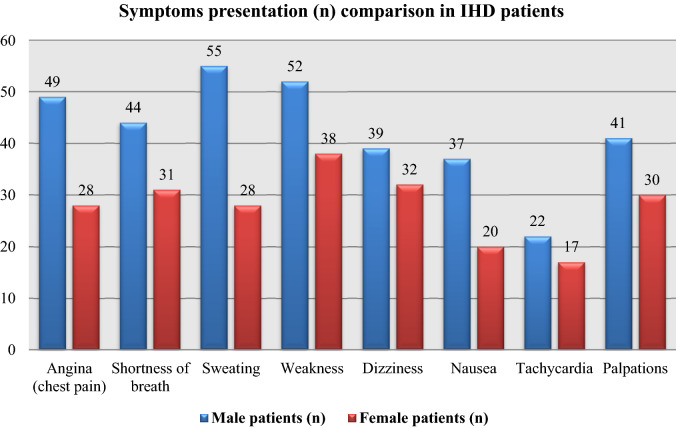
Depicts the presentation of symptoms (n) in IHD patients.

The symptoms detected in IHD male and female patients include sweating (90.2%; 71.8%), weakness (85.3%; 97.4%), angina (80.3%; 71.8%), shortness of breath (72.1%; 79.5%), dizziness (63.9%; 82.1%), and nausea (60.7%; 51.3%), respectively (Table 4 and Fig. 2). Table 4 also shows that symptoms such as dizziness, nausea, tachycardia, and palpation were significantly associated with IHD in male patients (*p* ≤ 0.05), whereas angina (chest pain), shortness of breath, sweating, weakness, dizziness, nausea, tachycardia, and palpation were significantly associated with IHD in female patients (*p* ≤ 0.05).

**Table 4:** Symptoms presentation (n; %) in IHD patients.

Patient symptoms	Male patients (n; %)	*P*-value	Female patients (n; %)	*P*-value
Angina (chest pain)	(49; 80.3%)	0.4602**	(28; 71.8%)	< 0.0001*
Shortness of breath	(44; 72.1%)	0.1356**	(31; 79.5%)	< 0.0001*
Sweating	(55; 90.2%)	0.1841**	(28; 71.8%)	< 0.0001*
Weakness	(52; 85.3%)	0.3822**	(38; 97.4%)	0.0105*
Dizziness	(39; 63.9%)	0.0176*	(32; 82.1%)	0.0002*
Nausea	(37; 60.7%)	0.0060*	(20; 51.3%)	< 0.0001*
Tachycardia	(22; 36.1%)	< 0.0001*	(17; 43.6%)	< 0.0001*
Palpations	(41; 67.2%)	0.0443*	(30; 76.9%)	< 0.0001*

*Significant correlation (*p* ≤ 0.05).

**No significant correlation (*p* > 0.05).

**Figure 3:**
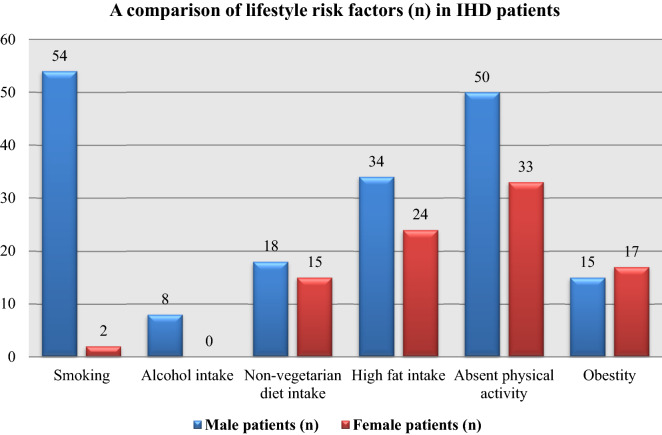
Depicts a comparison of lifestyle risk factors (n) in IHD patients.

Lifestyle risk factors of IHD patients in the current study were smoking (88.5%; 5.1%), absent physical activity (82%; 84.6%), high fat intake (55.7%; 61.5%), non-vegetarian diet intake (29.5%; 38.5%), obesity (24.6%; 43.6%), and alcohol intake (13.1%; 0%) in male and female patients, respectively (Table 5 and Fig. 3). Table 5 also shows that alcohol consumption, non-vegetarian diet consumption, high fat consumption, and obesity were all significant lifestyle risk factors for IHD in male patients (*p* ≤ 0.05). In female patients, smoking, non-vegetarian diet intake, high fat intake, lack of physical activity, and obesity were significant lifestyle risk factors for IHD (*p* ≤ 0.05).

**Table 5:** Lifestyle risk factors presented in IHD patients.

Lifestyle risk factors of IHD	Male patients (n; %)	*P*-value	Female patients (n; %)	*P*-value
Smoking	(54; 88.5%)	0.2421**	(2; 5.1%)	< 0.0001*
Alcohol intake	(8; 13.1%)	< 0.0001*	(0; 0%)	–
Non-vegetarian diet intake	(18; 29.5%)	< 0.0001*	(15; 38.5%)	< 0.0001*
High fat intake	(34; 55.7%)	0.0009*	(24; 61.5%)	< 0.0001*
Absent physical activity	(50; 82%)	0.5398**	(33; 84.6%)	0.0004*
Obesity	(15; 24.6%)	< 0.0001*	(17; 43.6%)	< 0.0001*

*Significant correlation (*p* ≤ 0.05).

**No significant correlation (*p* > 0.05).

**Figure 4:**
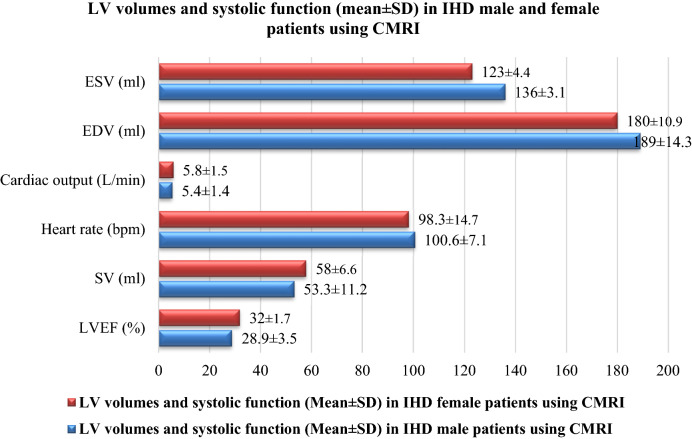
LV volumes and systolic function (mean ± SD) in IHD male and female patients using CMRI.

The LV volumes and systolic function by age decile in IHD male patients using CMRI were presented in Table 6. The mean ± SD was 28.9 ± 3.5%, 53.3 ± 11.2 ml, 100.6 ± 7.1 bpm, 5.4 ± 1.4 L/min, 189 ± 14.3 ml, and 136 ± 3.1 ml for LVEF, SV, heart rate, cardiac output, EDV, and ESV, respectively. In contrast, the LV volumes and systolic function by age decile in IHD, female patients using CMRI were presented in Table 7. In addition, the mean ± SD was 32 ± 1.7%, 58 ± 6.6 ml, 98.3 ± 14.7 bpm, 5.8 ± 1.5 L/min, 180 ± 10.9 ml, and 123 ± 4.4 ml for LVEF, SV, heart rate, cardiac output, EDV, and ESV, respectively. Figure 4 depicts the LV volumes and systolic function (mean ± SD) in male and female IHD patients who underwent CMRI.

**Table 6:** LV volumes and systolic function by age decile in IHD male patients using CMRI.

Males age ranges (years)	13–23	24–34	35–45	46–56	57–67	68–78	79–89	90–100	Mean ± SD
LVEF (%)	34.1 ± 6.1	30.5 ± 4.9	30 ± 4.1	29 ± 2.5	27.4 ± 3.2	25.6 ± 2.1	31.4 ± 5.5	22.8 ± 1.3	28.9 ± 3.5
SV (ml)	73 ± 19.3	61 ± 17.5	59 ± 15.4	55 ± 3.0	51 ± 0.2	46 ± 7.7	42 ± 6.5	39 ± 4.0	53.3 ± 11.2
Heart rate (bpm)	104.1 ± 11.0	114.8 ± 12.2	101.7 ± 8.2	92.7 ± 2.5	98 ± 6.4	93.5 ± 4.1	97.6 ± 4.9	102.6 ± 9.7	100.6 ± 7.1
Cardiac output (L/min)	7.6 ± 2.4	7 ± 2.1	6 ± 1.9	5.1 ± 1.6	5 ± 1.2	4.3 ± 0.9	4.1 ± 0.8	4 ± 0.5	5.4 ± 1.4
EDV (ml)	214 ± 24.6	200 ± 22.3	197 ± 19.6	190 ± 16.6	186 ± 13.0	180 ± 9.9	175 ± 8.3	171 ± 5.0	189 ± 14.3
ESV (ml)	141 ± 5.4	139 ± 4.9	138 ± 4.3	135 ± 3.7	135 ± 2.9	134 ± 2.2	133 ± 1.8	132 ± 1.1	136 ± 3.1

Left ventricle ejection fraction *LVEF*, Stroke volume *SV*, End diastolic volume *EDV*, End systolic volume *ESV.*

**Table 7:** LV volumes and systolic function by age decile in IHD female patients using CMRI.

Females age ranges (years)	13–23	24–34	35–45	46–56	57–67	68–78	79–89	90–100	Mean ± SD
LVEF (%)	35 ± 2.9	33.9 ± 2.7	32.1 ± 2.0	32.2 ± 2.3	31.6 ± 1.6	30.6 ± 1.0	30 ± 0.6	30.8 ± 1.2	32 ± 1.7
SV (ml)	70 ± 11.5	65 ± 10.4	59 ± 9.1	58 ± 7.7	56 ± 6.0	53 ± 4.6	51 ± 3.9	52 ± 2.4	58 ± 6.6
Heart rate (bpm)	114.3 ± 23.0	120 ± 25.5	108.5 ± 20.2	98.3 ± 17.1	92.9 ± 13.4	86.8 ± 10.2	86.3 ± 8.6	78.9 ± 5.2	98.3 ± 14.7
Cardiac output (L/min)	8 ± 2.6	7.8 ± 2.4	6.4 ± 2.1	5.7 ± 1.8	5.2 ± 1.4	4.6 ± 1.0	4.4 ± 0.9	4.1 ± 0.5	5.8 ± 1.5
EDV (ml)	200 ± 18.9	192 ± 17.1	184 ± 15.0	180 ± 12.7	177 ± 10.0	173 ± 7.6	170 ± 6.4	169 ± 3.9	180 ± 10.9
ESV (ml)	130 ± 7.6	127 ± 6.8	125 ± 6.0	122 ± 5.1	121 ± 4.0	120 ± 3.0	119 ± 2.5	117 ± 1.6	123 ± 4.4

Left ventricle ejection fraction *LVEF*, Stroke volume *SV*, End diastolic volume *EDV*, End systolic volume *ESV.*

Diagnostic test characteristics revealed that CMRI in the diagnosis of IHD in the patients has a sensitivity, specificity, and accuracy of 97%, 33.33%, and 95.15%, respectively. In addition, the positive and negative likelihood ratio for CMRI in the diagnosis of IHD were 1.45 and 0.09. Furthermore, the disease prevalence was determined to be 97.09% by using invasive coronary angiography. PPV and NPV of CMRI in the diagnosis of IHD in the patients were 97.98% and 25%, respectively (Table 8). Much more, the 95% confidence interval (CI), which is a type of estimate computed from the statistics of the observed data, was determined for the diagnostic test characteristics (Table 8). The two female patients who were excluded from the study samples shared the following characteristics: They were 24 and 27 years old, and they were suffering from dizziness, nausea, and tachycardia. Smoking, a non-vegetarian diet, a high fat intake, a lack of physical activity, and obesity were among their lifestyle risk factors for IHD. They were also the CMRI false positive cases who were diagnosed with IHD, but this error was corrected by invasive coronary angiography, proving they were not affected by IHD and thus excluded from the study samples. Finally, after undergoing CMRI and invasive coronary angiography to confirm the absence of IHD, one true negative case was excluded from the study samples. His characteristics traits were as follows: Shortness of breath and palpations were the accompanying symptoms in a 37-year-old male patient, and lifestyle risk factors for IHD included smoking, alcohol consumption, and a lack of physical activity (Table 8).

**Table 8:** Diagnostic test characteristics of CMRI in the diagnosis of IHD in the patients.

Test	Present	n
Positive	True positive	97
Negative	False negative	3

Test	Absent	n
Positive	False positive	2
Negative	True negative	1

Statistics	Value	95% confidence interval (CI)
Sensitivity	97%	91.48% to 99.38%
Specificity	33.33%	0.84% to 90.57%
Positive likelihood ratio	1.45	0.65 to 3.24
Negative likelihood ratio	0.09	0.01 to 0.63
Disease prevalence (using invasive coronary angiography)	97.09%	91.72% to 99.40%
Positive predictive value (PPV)	97.98%	95.61% to 99.08%
Negative predictive value (NPV)	25%	4.53% to 70.90%
Accuracy	95.15%	89.03% to 98.41%

## Discussion

Several comparative studies have been published to evaluate CMRI as a diagnostic tool for CAD using conventional angiography as the gold standard^9,14,15^. According to the findings of these studies, CMRI is an accurate imaging tool for detecting myocardial viability^9^. For several years, CMRI has been used in clinical settings to measure LV volume, systolic function, and mass using standardized short axis multi-slice acquisition methods^14^. CMRI’s excellent accuracy and reproducibility are well established, establishing it as a gold standard technique that can be very cost effective^14,15^.

In the current study, the results of the CMRI echo were compared to those of the corresponding conventional coronary angiograms. Conventional invasive coronary angiography is the gold standard for the diagnosing the presence of significant stenosis; it is reserved for patients with a high clinical risk or when stress testing reveals a significant ischemic burden^16^. This study aimed to investigate the value of using CMRI as a diagnostic tool to diagnose IHD in a sample of 100 Saudi patients who were clinically diagnosed with IHD and recruited prospectively at the Radiology and Medical Imaging Department, KFMC, Riyadh- Saudi Arabia (Table 3).

To the best of our knowledge, the current study is the first study in Saudi population to determine the CMRI results in IHD patients. These findings could be compared to those of a study on normalized LV systolic and diastolic function using steady state free precession cardiovascular magnetic resonance^13^.

The effect of gender on LV volume reveals that males have significantly larger LV volumes (*p* < 0.001)^13^. Gender had a significant independent influence on normalized LV volume in a multivariate analysis^13^. In terms of the effect of age on LV parameters, females had a significant decrease in absolute and normalized ESV with increasing age (*p* = 0.013)^13^. Normalized EDV decreased with age in both men and women (*p* = 0.019)^13^. LVEF did not change significantly with age in either men or women. Age was found to be an independent predictor of all absolute and normalized ventricular volumes in a multivariate analysis (EDV, ESV, and SV). With age, both absolute and normalized ventricular volumes decreased significantly^13^.These findings could be compared with our results on age-decile LV volumes and systolic function in IHD male and female patients using CMRI, which were presented in (Tables 6 and 7).

One of the most significant strengths of this study is that it used a diagnostic test to reveal the sensitivity, specificity, and accuracy of CMRI in the diagnosis of IHD in Saudi patients, after each case had been confirmed using conventional angiography as the gold standard tool. Whereas it has been statistically proven that CMRI had a sensitivity, specificity, and accuracy in the diagnosis of IHD (Table 8) which is a benefit for the current study based on that this finding regarding sensitivity, specificity, and accuracy is not available in comparable studies for comparison. The limitations of this study were the small cohort sample size and population heterogeneity, which could affect the accuracy of our findings and the intensity of our conclusions, as it allows other age groups to have less objective value if committed in subsequent inquiries. To the best of our knowledge, however, this is the first cohort study determine the use of CMRI as a diagnostic method in in Saudi population with IHD, highlighting the significance of this study.

In conclusion, CMRI provides a comprehensive assessment of LV function, myocardial perfusion and viability, and as well as the coronary anatomy. One of the most significant strengths of this study is that it revealed the sensitivity, specificity, and accuracy of CMRI in the diagnosis of IHD in the patients and that was after the confirmation of the diagnosis of each case was confirmed using the conventional angiography as the gold standard tool.

## Data Availability

The datasets generated during and/or analysed during the current study are available from the corresponding author on reasonable request.
